# Enhancing DDoS detection in SDIoT through effective feature selection with SMOTE-ENN

**DOI:** 10.1371/journal.pone.0309682

**Published:** 2024-10-17

**Authors:** Arati Behera, Kshira Sagar Sahoo, Tapas Kumara Mishra, Anand Nayyar, Muhammad Bilal

**Affiliations:** 1 Department of Computer Science and Engineering, SRM University, Amaravati, Andhra Pradesh, India; 2 Department of Computing Science, Umeå University, Umeå, Sweden; 3 Faculty Information Technology, Duy Tan University, Da Nang, Vietnam; 4 School of Computing and Communications, Lancaster University, Bailrigg, Lancaster, United Kingdom; St Xavier’s Catholic College of Engineering, INDIA

## Abstract

Internet of things (IoT) facilitates a variety of heterogeneous devices to be enabled with network connectivity via various network architectures to gather and exchange real-time information. On the other hand, the rise of IoT creates Distributed Denial of Services (DDoS) like security threats. The recent advancement of Software Defined-Internet of Things (SDIoT) architecture can provide better security solutions compared to the conventional networking approaches. Moreover, limited computing resources and heterogeneous network protocols are major challenges in the SDIoT ecosystem. Given these circumstances, it is essential to design a low-cost DDoS attack classifier. The current study aims to employ an improved feature selection (FS) technique which determines the most relevant features that can improve the detection rate and reduce the training time. At first, to overcome the data imbalance problem, Edited Nearest Neighbor-based Synthetic Minority Oversampling (SMOTE-ENN) was exploited. The study proposes SFMI, an FS method that combines Sequential Feature Selection (SFE) and Mutual Information (MI) techniques. The top *k* common features were extracted from the nominated features based on SFE and MI. Further, Principal component analysis (PCA) is employed to address multicollinearity issues in the dataset. Comprehensive experiments have been conducted on two benchmark datasets such as the KDDCup99, CIC IoT-2023 datasets. For classification purposes, Decision Tree, K-Nearest Neighbor, Gaussian Naive Bayes, Random Forest (RF), and Multilayer Perceptron classifiers were employed. The experimental results quantitatively demonstrate that the proposed SMOTE-ENN+SFMI+PCA with RF classifier achieves 99.97% accuracy and 99.39% precision with 10 features.

## 1 Introduction

The Internet of Things (IoT) infrastructure is constantly expanding, and already has billions of physically connected gadgets [1, 2]. Due to the heterogeneity characteristics of the devices, they have severe security flaws. As a consequence, the prevalence of the IoT has greatly increased the number of cyber attacks globally [3]. IoT networks have several characteristics, including scalability, availability, efficiency, and reliability. As the size of the network grows, the above-mentioned constraint should be maintained. Software-defined networks (SDN) increase the flexibility and adaptability of networks. IoT networking is an architecture that efficiently abstracts numerous network layers [4, 5]. By enabling businesses and service providers to react quickly to shifting business requirements, SDN aims to enhance network control. IoT network with the supervision of SDN called SDIoT, assists in resource management and keep of network functionalities without compromising network performance [6].

Cyber attacks have recently targeted several IoT networks; for instance, on October 21, 2016, a DDoS attack utilizing Mirai’s Botnet impacted Dyn Server, a corporation that manages a large portion of the Internet DNS infrastructure in America [7]. Major websites like Amazon, Netflix, Spotify, PayPal, and Twitter in the US and Europe are affected by these attacks. This attack was discovered by cybersecurity researchers at Trend Micro and was affecting 122,069 IP cameras across the globe [8]. Recent cyber attacks on IoT networks have caused significant device damage, which served as the motivation for our study. To protect from sophisticated cyberattacks, it is on the top of the priority to quickly deploy intelligent solutions in IoT-based applications. Therefore, SDIoT-like new approaches need to be acknowledged for the DDoS attacks problems.

In the SDIoT, all IoT-enabled devices including smart watches, smartphones, smart hospitals, and smart vehicles constantly generate a large volume of data from the end users. All these heterogeneous services must be supported within a common architecture. According to the International Telecommunication Union Telecommunication Standardization Sector (ITU-2015) [9], the architecture of an IoT system should be organized into three layers: a) a sensing layer, containing all essential protocols for implementing data sensing units in IoT applications; b) a network layer, responsible for supporting all communication technologies; and c) an application layer, incorporating application support services, data service APIs, etc.

**Fig 1:**
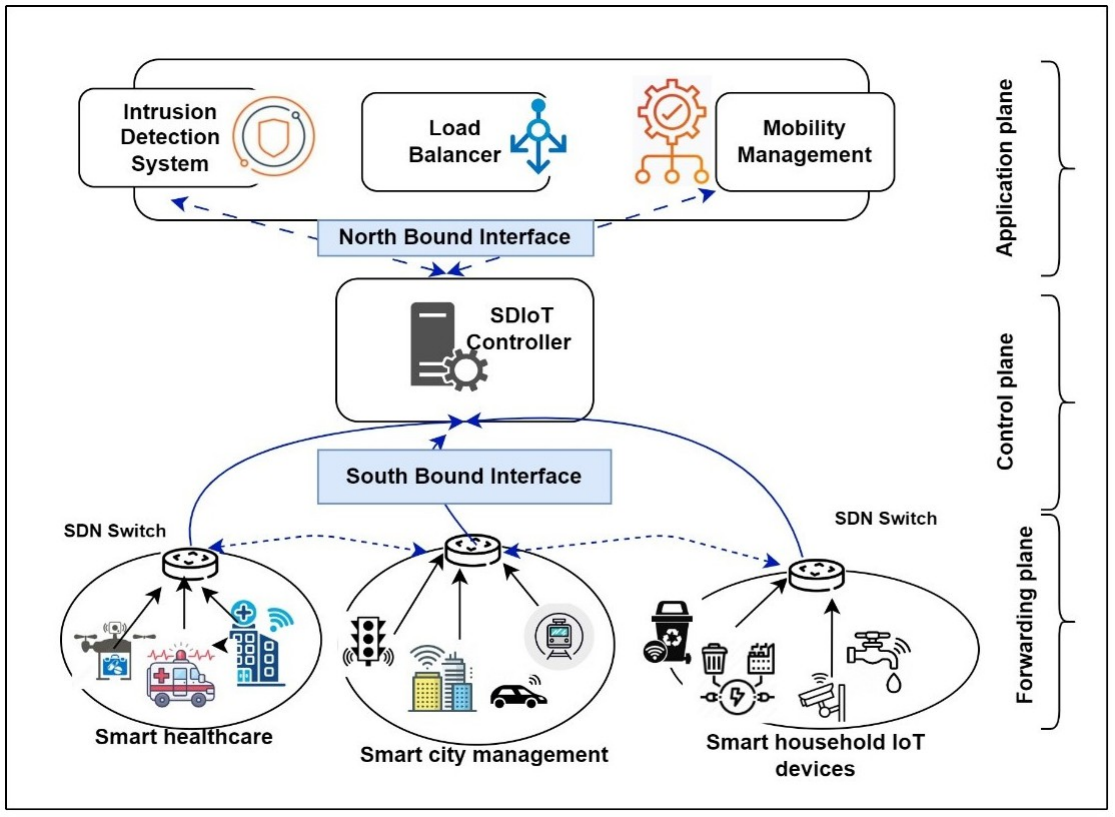
A typical SDIoT scenario.

Fig 1 demonstrates an SDIoT scenario which has been inspired by work presented in [10], where authors introduced an energy-aware architecture utilizing a decentralized approach with blockchain and SDIoT scenarios to address various issues faced by smart society.

As demonstrated in the figure, a communication network is formed by all switches, and they act as the forwarding plane of the SDN network. This plane is responsible for forwarding the data from the IoT devices. The SDIoT controller manages each base station and is responsible for maintaining the security of the entire communication network. Most of the anomaly detection solutions for SDN have used machine learning (ML) and knowledge-based techniques to identify the attack [9–11]. The success of the ML classifiers sincerely depends on how accurately the anomaly traffic is detected [12]. In a real-time network, selecting key features from the data is important to make the classifier more sophisticated and effective for the identification of malicious attacks. To effectively reduce the amount of data, feature selection techniques can be employed in data pre-processing [13, 14].

The feature selections are of three categories such as filter method, wrapper method, and embedded method [15]. Here, the authors have implemented the wrapper methods. This method can remove noise data, and redundant and less important features from the dataset. Improved feature selection mechanism enhances both execution speed and accuracy [16]. As a result, the developer can construct effective models to detect DDoS attacks in real-time networks with fewer computational resources and prediction latency. Like feature selection, various feature reduction techniques such as principal component analysis (PCA), Linear Discriminant Analysis (LDA), and multidimensional scaling can be used for data preprocessing. These methods transform the original features into a new set of features [17].

This study suggests the following contributions, taking into account the necessity for advanced DDoS classification with low latency capability in an SDIoT network environment. The major contribution of this work can be summarized as follows:

- This work aims to develop a lightweight multi-class DDoS attack solution using improved FS selection techniques and ML algorithms. The feature selection method is employed to improve the intrusion detection rate and reduce the training time.
- To overcome the imbalance nature and over-sampling bias in the dataset, SMOTE- Edited Nearest Neighbor (ENN) is being utilized.
- The balanced data is used as an input for the wrapper-based FS technique and filter-based FS technique separately. The study proposes SFMI that combines the advantages of both SFE and Mutual Information techniques. Top *k* common features were extracted from the nominated features based on SFE and MI. Further, PCA is employed to address multi-collinearity and redundancy issues.
- For classification purposes, five different classifiers such as Decision Tree, K-Nearest Neighbour, Gaussian Naive Bayes, Random Forest, and Multilayer Perceptron are employed.
- The performance evaluation was conducted on benchmark datasets such as: KDDCup99 and the recently released CIC IoT-2023 dataset. A multiclass comparison and computational complexity analysis were made on the test dataset.

### 1.1 Background

Most of the anomaly detection solutions for SDN have used machine learning and knowledge-based techniques to identify the attack [18]. The success of the classifiers depends on how accurately they can predict the attack traffic. In a real-time network, timely measurements are the key factor. As a result, selecting prime features from the data is important to make the classifier more sophisticated and effective for the identification of attack traffic [12]. The SDN controller handles all application communication and networking equipment. Due to northbound interfaces, the controller will communicate with applications such as network monitoring, flow management, network management, firewall, load balancer services, and DDoS attack detection systems [19]. A southbound interface, such as the SDN OpenFlow protocol, enables the controller to communicate with particular network devices in the data plane [20, 21]. Using these southbound protocols, the controller can arrange the network devices and select the optimal network connectivity route for application traffic. In research, there are many datasets used for DDoS attack prediction, here the author used the KDDCup99 dataset for predicting DDoS attacks with SDIoT [22]. Various Machine Learning and Deep Learning based algorithms for predicting DDoS attacks were developed in the given context. The problem of an imbalanced dataset is not taken into consideration by many researchers, who instead concentrate on feature selection strategies and classification algorithms [23]. The accuracy of the classification algorithm is greatly affected by the issue of class imbalance. Additionally, a lot of attributes are needed for prediction when the data is unbalanced. This certainly makes the solution computationally complex, making it unusable in a real-world situation. This considerably increases the computing complexity of the solution, rendering it unsuitable for use in a real-world setting [24].

Furthermore, in order to decrease computing while maintaining reasonable accuracy, it is necessary to update the current feature selection techniques. Similarly, to this, improved classifier results are required to generate reliable outcomes. In summary, the prediction of attack traffic in a real network like SDIoT unified machine learning technique is needed. To add this data balancing, feature selection, and classification improvement need to be carried out systematically.

The rest of the paper is divided into five sections. Section 2 briefly discusses the pre-existing research work and briefly provides theoretical and mathematical explanations about DDoS and ML techniques. In section 3, the authors have discussed the proposed methodology, datasets and PCA, and various feature selection methods. Section 4 contains the result analysis and presents the evaluation. Section 5 concludes the paper with future scope.

## 2 Related work

In this section, we briefly discuss existing research about feature selection for DDoS attack detection in both SDN and IoT Networks.

Using various data sets and approaches, researchers have suggested multiple algorithms in order to predict DDoS attack Detection with networks. Razan et al. [23] proposed a multi-class combined performance metrics concerning class distribution to compare various multi-class and binary classifications. They used an auto encoder to assign the values to categorical data and PCA to reduce the dimensions. Aljawarnch et al. [25] proposed an anomaly based intrusion detection system through feature selection analysis hybrid feature selection method using correlation-based feature selection and information gain. They applied adaptive boosting using Naive Bayes as the weak classifier. Here correlation is done using greedy search and classifier on the reduced NSL-KDD dataset. In another work, Zong et al. [19] proposed the combination of matrix diversity and PCA for DDoS and feature reduction. They demonstrated a higher prediction accuracy than the traditional method. In this work, the authors used the KDDCup99 dataset. In [26] authors proposed a multi-objective optimization-based feature selection method for the detection of anomaly traffic in IoT. They have implemented the multi-objective evolutionary algorithm with an adapted jumping gene operator. They exploited an Extreme Learning machine (ELM) as the classifier for feature selection based on six critical objectives for an IoT network. The PCA was used to reduce the dimension of the dataset from a large number of features to a small number by Shengchu Zhao et al. [27]. For classification purposes authors used Softmax Regression and K-nearest neighbor algorithms. Softmax Regression achieves better accuracy using the KDDCup99 dataset. Panda et al. [28] suggested semi-naive Bayesian, Decision Tree-based, Chi-square automatic interaction identification. Next, a hybrid genetic algorithm and K-mean clustering were utilized, along with two dependency estimators. Deep Multi-Layer Perceptron and Convolutional-Neural Network based classifiers are two instances of deep learning techniques used [29]. Further in [30], two features selecting methods i.e. information gain and RF analysis are used by the authors. For improving accuracy, deep learning techniques and LSTM and Autoencoder were used to solve the issue of DDoS attacks in SDNs. In [31], the authors approached the feature selection method Extreme Gradient Boosting for determining the most relevant features with a hybrid Convolutional Neural Network and Long-Short Term Memory (CNN-LSTM) for DDoS attack classification. The proposed model applied on the CICIDS2019 dataset with improved accuracy. In a similar work, Abubakr et al. [32] used a wrapper method for feature selection using a binary-particle swarm optimization algorithm and the Decision Tree approach.

Brao et al. explored variance indexing methods using a feature selection algorithm for intrusion detection [33]. They specified the KNN method to improve partial distance search and different types of classification for the significance implemented on the NSL-KDD dataset. An ensemble framework (EnFs) has been proposed by Das et al. [34]. The framework combines the outputs of seven important features using the majority voting technique and produces an optimal set of features on the NSL-KDD dataset. In [35] researchers combined the information gain, PCA with an ensemble classifier and SVM instance-based learning algorithms over ISCX2012, NSL-KDD, and Kyoto-2006 datasets. In a different context, Bawany et al. [16] employed SEAL, an SDN-based adaptive framework, for protecting smart city applications against DDoS attacks. Chen et al. [36] proposed a statistical-based trace-back scheme using the SDN architecture. They have analyzed the changes of network flow through the base station and multiple controllers. In [37], authors proposed a novel feature selection approach for the network intrusion detection system in a cloud environment. Authors in [38], suggested an ensemble based multi-feature selection method that combines the output of four filter methods to achieve an optimum selection using intrusion detection. In [39], authors suggested a FS method which is based on mutual information. In [40], authors proposed PCA as the FS and SVM as the classifier for their anomaly detection work. Lin et al. [41] prosed BFE for their work.

From the above literature survey, it can be observed that the DDoS detection using machine learning techniques has not been well explored in the SDIoT like modern network infrastructure. Usually, the anomaly datasets are imbalanced in nature and in this direction limited research has been carried out. To overcome the data imbalance, the framework suggests utilizing the SMOTE technique. So far, many research were conducted the experiments with binary classes out of 23 types of attacks. Due to the higher execution time, previous researchers were unable to cover all the classes. For this reason, the accuracy and detection rate vary. However, the current study attempts to include 11 types of attacks as four classes.

## 3 Materials and methods

The following section discuss about the dataset, feature selection, feature reduction, and various ML models used in this work.

### 3.1 Materials

The Subsequent section discusses the dataset, various data pre-processing techniques opted in the work.

#### 3.1.1 Dataset

There are multiple datasets used in research for the prediction of DDoS attacks. The authors used the KDDCup99 dataset for the prediction of DDoS attacks. The KDDCup99 dataset is widely used in IoT and SDN frameworks for DDoS attack detection using ML for training purposes because it is a well-known and well-established dataset in the field of network security [24]. The dataset provides a large set of labeled network traffic data that can be used to train machine learning algorithms to detect various types of network attacks, including DDoS attacks. Additionally, the use of this dataset helps to ensure that the resulting ML models are not overfitting to a specific dataset, and it mimics real-world network traffic data.

Additionally, we employed the CIC IoT-2023 dataset, a new and extensive resource that provides unique benefits and expands upon earlier datasets [42]. The latest dataset, released by the Canadian Institute for Cybersecurity, is created specifically for security analytics applications for real-time IoT operations. This dataset introduces a unique and comprehensive compilation of IoT attack data having 47 features including target value with 238687 instances, featuring 34 attack classes conducted in an IoT topology consisting of 105 devices.

#### 3.1.2 Data pre-processing

There are many methods are available for data pre-processing. For data pre-processing authors address the threes issues such as (i) Handling null values (ii) Standardization (iii) Handling categorical values. The detailed process is discussed below.

1. **Handling Null Values** In the KDDCup99 and CIC-IoT2023 dataset few features contain null values. This issue is handled by dropping rows.
2. **Standardization** In this study, feature scaling is accomplished using the standardization technique, in which the values are updated to the mean with a standard deviation of one unit. Thus, the required columns are updated using Eq 1.
  $$\begin{array}{c} x^{\prime }=\frac{\left(x-\mu \right)}{\sigma } \end{array}$$ (1)
  where *μ* is the mean of the feature values and *σ* is the standard deviation of the feature values. Standardization is helpful in cases where the data follows a Gaussian distribution for standardization of the data set, which is discussed in Eq 2.
  $$\begin{array}{c} P\left(\frac{x_{i}}{y_{c}}\right)=\frac{1}{\sqrt{2\pi \sigma^{2}y_{c}}}exp^{(}\frac{-{\left(x_{i}-\mu y\right)}^{2}}{2\mu y_{c}^{2}}) \end{array}$$ (2)
3. **Handling Categorical values** In the data set, four categorical variables are present such as protocols, service, flag, and label. For handling categorical values we used the label encoder technique. This data can be replaced with 1, 22, 9, and 3 respectively.

#### 3.1.3 Class selection in both dataset

We have categorized the different attacks to verify the effectiveness of the feature selection methods. Eleven out of 23 different types of attacks have been selected. These 11 types of attacks were divided into 4 different classes *DOS*, *Probe*, *R2L*, and *Normal*.

The CIC-IoT dataset has 34 classes and every attack carried out for this study has a unique set of features. We converted 34 classes into 7 classes i.e.; DDoS, DoS, Recon, Web-based, Brute Force, Spoofing, Mirai and Normal attack. Each category and categories are listed in Table 1.

**Table 1:** Names of each attack and category.

Names	Attacks
DDoS	ACK Fragmentation
	UDP Flood
	SlowLoris
	ICMP Flood
	RSTFIN Flood
	PSHACK Flood
	HTTP Flood
	UDP Fragmentation
	ICMP Fragmentation
	TCP Flood
	SYN Flood
	SynonymousIP Flood
DoS	TCP Flood, HTTP Flood,
	SYN Flood, UDP Flood
Recon	Ping Sweep, OS Scan, Host Discovery Vulnerability Scan, Port Scan,
Web-Based	Sql Injection, Command Injection, Backdoor Malware, Uploading Attack, XSS, Browser Hijacking
Brute Force	Dictionary Brute Force
Spoofing	Arp Spoofing, DNS Spoofing
Mirai	GREIP Flood, Greeth Flood, UDPPlain

Table 1 provides a thorough summary of several cyberattacks, divided into sections for Denial-of-Service (DoS), Distributed Denial-of-Service (DDoS), Vulnerability Scan, Brute Force, Spoofing, and Internet of Things Malware attacks. Attack methods including ACK Fragmentation, UDP Flood, Ping Sweep, OS Scan, Sql Injection, Dictionary Brute Force, Arp Spoofing, Mirai, and GREIP Flood are all included in each category.

### 3.2 Methods

Subsequent section discusses the ML methods, dimension reduction and feature selection methods employed in the work.

#### 3.2.1 SMOTE

SMOTE generates new artificial instances using information about the neighbors that surround each sample in the minority class [22, 43, 44]. SMOTE creates synthetic training cases for the minority class using linear interpolation [45] These synthetic training cases are constructed by randomly selecting a subset of the k-nearest neighbors for each instance in the minority class [46].

**Fig 2:**
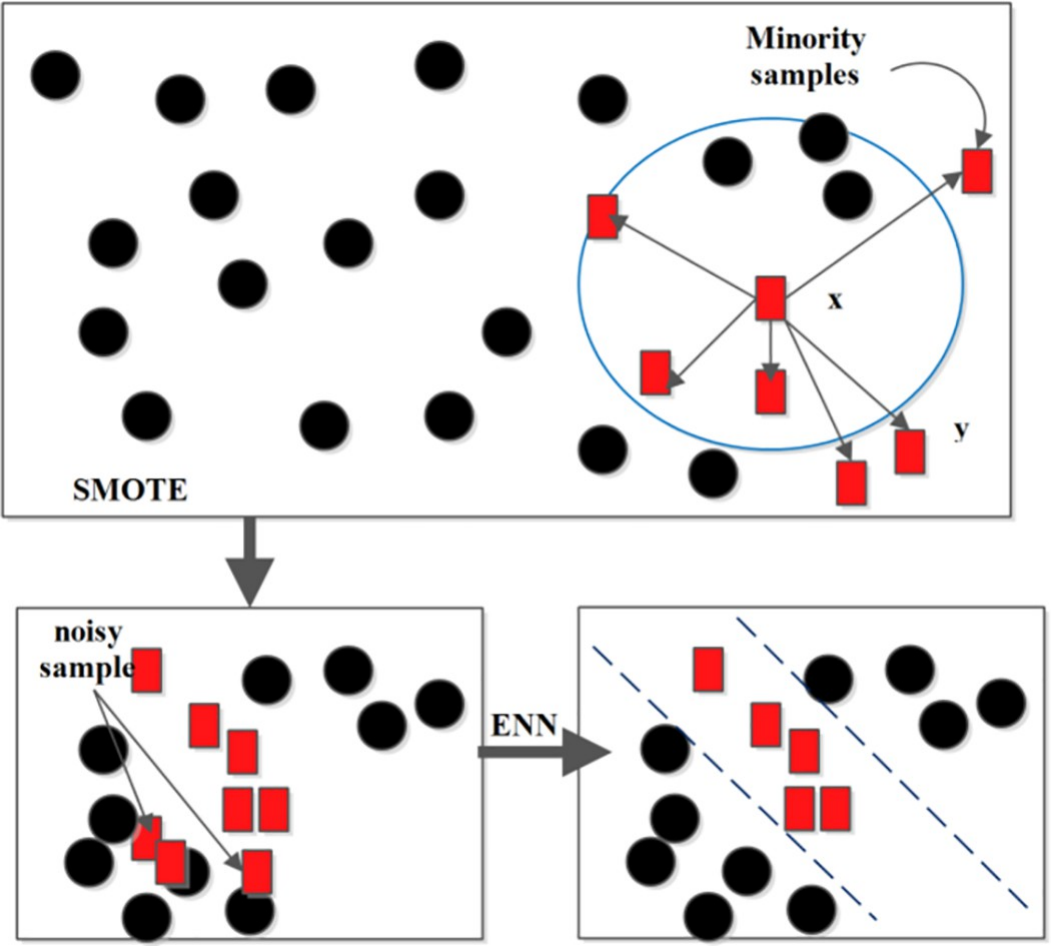
Process of SMOTE during synthesis and eliminating noisy samples using ENN.

However, we have explored Edited Nearest Neighbor with SMOTE (SMOTE-ENN). SMOTE-ENN combines the SMOTE oversampling technique with an undersampling technique called ENN. It separates the presence of any noisy or borderline samples from the dataset by considering their class label against their K-Nearest Neighbors (K-NN). In case the class labels do not equal, then the sample is considered as noisy and then both observation and its KNN are removed. SMOTE selects each minority sample as the root sample for synthesis of the new sample. Further ENN eliminates noisy samples whose most KNN samples are different from other classes which is illustrated in Fig 2. The step-by-step process of SMOTE-ENN is discussed in Algorithm 2.

#### 3.2.2 Feature selection

Feature selection is the process of selecting a subset of the original characteristics to reduce model complexity, improve computing performance, and reduce generalization error caused by noise provided by irrelevant features [20]. In this study, two feature selection methods have been examined. These methods can be used in the proposed framework’s feature engineering modules. First one is the sequential feature selection and the second one is backward feature elimination which comes under the wrapper method [15]. The wrapper approach is the primary focus of this paper. The wrapper techniques employ a search strategy to combine the space of potential feature subsets and rank them accordingly [47]. The wrapper technique is based on greedy search algorithms, which consider all feasible feature combinations and produce the best outcomes [34]. Feature selection techniques which include sequential feature selection (SFS), backward feature elimination (BFE), recursive feature selection, exhaustive feature selection, etc. are the most popular approach categories. In this paper, we have explored SFS and BFE methods.

1. Sequential Feature Selection
  The SFE technique is a family of greedy search techniques, that reduces an initial d-dimensional feature space to a k-dimensional feature subspace, where k<d. Finding the ideal subset of features in λ is the process referred to as feature selection. Thus, the problem can be expressed as, {*Y*(λ): λ ∈ 2^*k*^},
  Where $\lambda \subset X_{i}^{j}$ is any subset of features, $2^{k}=\left\{\lambda :\lambda \subset X_{i}^{j}\right\}$ is the set of feasible solution and *Y*(λ) is objective function used to measure of quality of λ. In this context, we search for a subset using SFS as an objective value.
2. Backward Feature Elimination
  Using a feature selection strategy, the traits that have no observable effect on the dependent variable or output prediction are eliminated [43]. As other features are added, the model grows increasingly complicated. Consequently, in order to get the best outcomes, it is essential to keep the model straightforward and to focus just on its most crucial components. This strategy is used to enhance the performance of the ML model by only including the features that have the greatest impact and eliminating the features that have the least impact.
3. Mutual Information
  Mutual information measures the amount of information obtained about one variable through the observation of another variable. MI value zero indicates two variables are independent. In FS, mutual information is used to evaluate the relevance of each feature to the target variable. A high MI score indicates that a feature provides valuable information about the target variable. A low score suggests that the feature is less informative about the target variable and may be considered for removal, which is discussed in Eq 3. The MI between two features (x) and (y) can be calculated as follows:
  $$\begin{array}{c} I\left(x,y\right)=\sum_{y^{\prime }\in y}\sum_{x^{\prime }\in x}P\left(x^{\prime },y^{\prime }\right)\text{log}(\frac{p(x^{\prime },y^{\prime })}{p\left(x^{\prime }\right)p\left(y^{\prime }\right)}) \end{array}$$ (3)

where, *I*(*x*, *y*) represents the MI between x and y. *P*(*x*′, *y*′) denotes the joint probability. P(*x*′) and P(*y*′) are the marginal probabilities.

#### 3.2.3 Classification techniques

- **Decision Tree**:
  The selection of attributes for root nodes within every level is the most difficult task in a Decision Tree. There are two popular methods for selecting attributes [48]. In machine learning, the DT algorithm works with no attribute-based parameter technique. If there is a single attribute that really can simply segregate data and improve decision-making, it works well. The range of the root node poses a hurdle in this approach. When the root node is chosen carefully, the algorithm’s computational complexity is reduced, and it becomes extremely effective.
- **Random Forest** It is a popular classifier for supervised learning. The key benefits are reduction of over-fitting, a shorter training period, and excellent accuracy.
- **Gaussian Naïve Bayes**:
  In Gaussian Naïve Bayes (GNB) a special type of Naive Bayes classifier. It is specially used in dataset features that have continuous value, then features are assumed to be Gaussian distribution and we call another name the normal distribution [49].
- **Multilayer Perceptron**:
  The multi-layer perceptron is an infinite sized directed acyclic graph. A decent generalization is the most widely used neural network architecture. The trained model can provide reliable output for the label and untested inputs. The early stopping criteria of the MLP classifier gives an approximation of the number of iterations that can be performed before the model becomes overfit [50].
- **K-Nearest Neighbour**: One of the most fundamental adaptive algorithms being used in supervised learning is the K-NN approach. In supervised learning, the training data is labeled and found unknown samples, the model forecasts it using a trained model [51]. KNN performs effectively on datasets with just many samples. It works well with numeric properties only. A distance metric is used to identify which of the K examples inside the training data are closest to the new input. Euclidean distance is the widely used distance measure for input variables with real values. The distance is measured using Eq 4.
  $$\begin{array}{c} ED\left(a,b\right)=\sqrt{\sum {\left(a-b\right)}^{2}} \end{array}$$ (4)
- **Principal Component Analysis (PCA)**:
  It is an unsupervised learning algorithm used in machine learning for dimensionality reduction [40]. The goal of PCA is to find a new set of orthogonal axes known as “principal components” that capture the most variance in the data. PCA is a widely used method for exploratory data analysis, as it can help identify patterns and relationships in high-dimensional datasets. Additionally, by reducing the number of dimensions, PCA can also improve the performance of machine learning algorithms that require a lower number of features.

## 4 Proposed model

**Fig 3:**
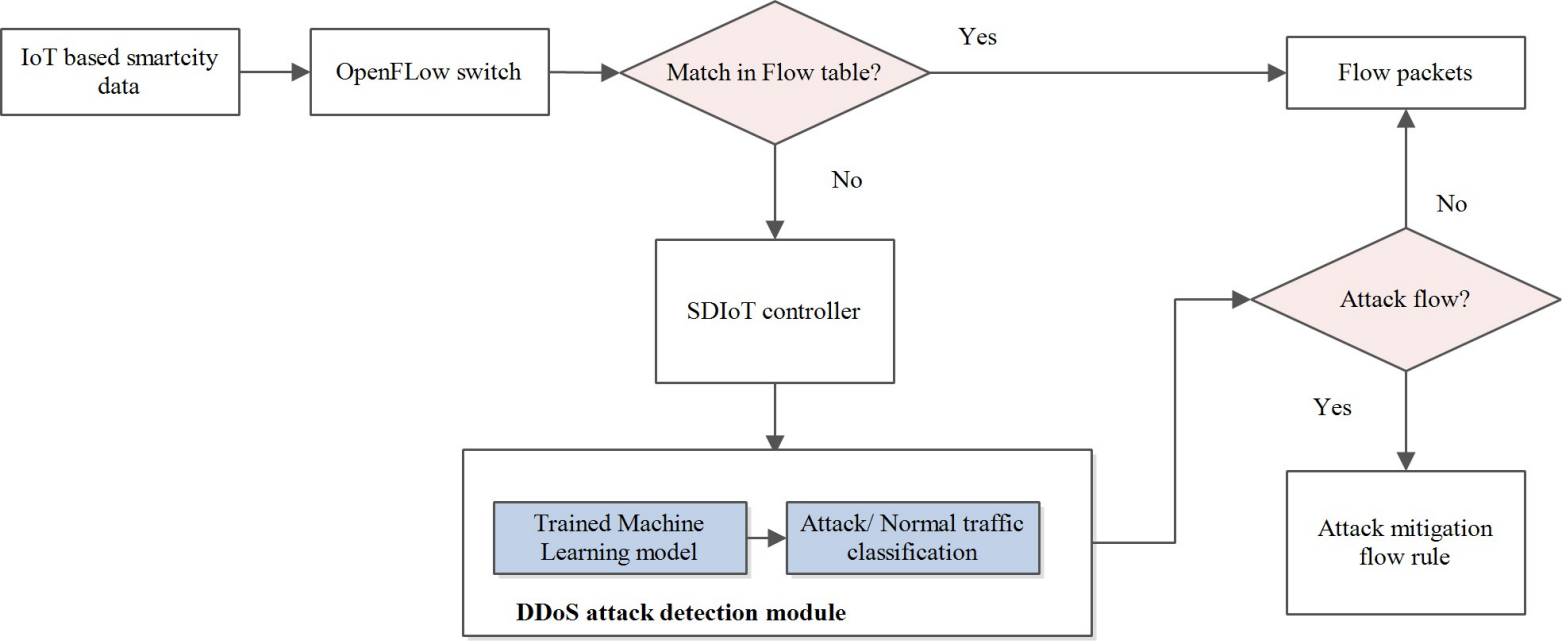
High-level design of anomaly detection framework in SDIoT.

Fig 3 shows the high level design of ML based anomaly detection framework utilizing SD-IoT. The detection scheme primarily consists of three main components: IoT devices, SDN switches, and SDIoT controllers. The major modules are residing in the SDN controller. It includes a feature extraction module, learning with detection module, and a flow management module. By using the OpenFlow protocol, the controller separates policies into service-specific rules and pushes them into the flow tables of the SDN switches [52]. Then the packet is forwarded based on these rules in the flow table. There are different fields that are stored in the flow table against each flow entry. Whenever a new packet arrives, it is matched with the flow table rules, in case of a match the controller takes the necessary action stored in the action field and in turn updates the counters. In case of a mismatch, a new rule is supplied to the flow table.

**Fig 4:**
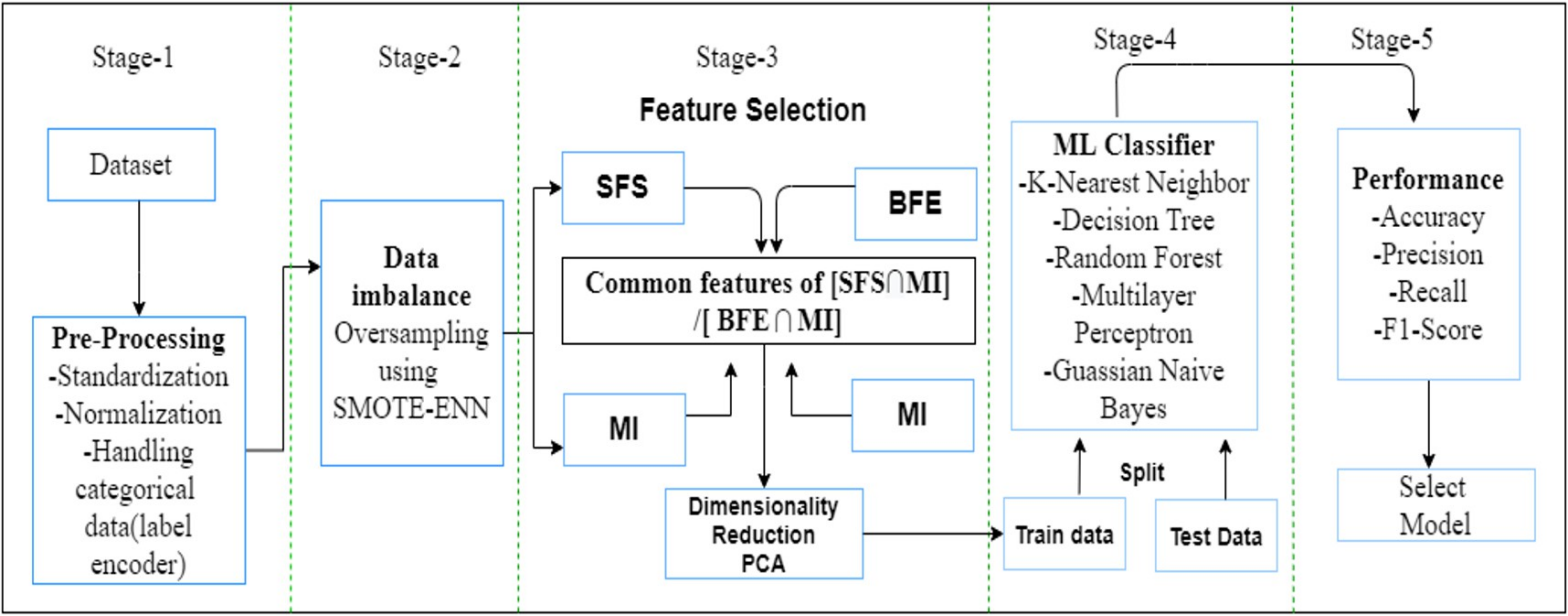
Flow diagram of the ML-based anomaly detection model.

The actions starting from pre-processing to classifier selection in the controller are segregated into five stages. The various steps used in this model are depicted in Fig 4. In the first stage prepossessing of the dataset is carried out. In this study, the KDDCup99 and CIC-IoT 2023 dataset were employed. This dataset needs to be balanced in the second phase since it consists of unbalanced classes. The feature selection technique was applied in the third phase to determine the reduced features and important features. The balanced data is used as an input for the wrapper-based SFS technique and filter based Mutual Information technique separately. The FS process called SFMI combines the advantages of both SFE and MI techniques. In SFMI each input feature is added to the final selected features set based on maximizing mutual information between selected inputs and target value. Here, MI helps to measure the goodness of the feature. On the other hand, SFE iteratively build the best performing feature subset for the predictive model. Further the top *k* common features were extracted from the nominated features based on SFE and MI.

Algorithm 1 summarizes the proposed approach.

**Algorithm 1** Proposed Anomaly Detection Model

1: Read the data set ND with features *x*_1_, *x*_2_, …, *x*_42_

2: Detect the attack traffic

3: Initialize Data ← ND

4: Pre-processing of the data set involves categorical value, Standardization, and Null values.

5: Balancing the data set using SMOTE-ENN ← $X_{i}^{j}$

6: FS1 ← Nominated feature set based on SFS

7: FS2 ← Nominated feature set based on MI

8: Top *k* (feature new) ← FS1 ∩ FS2

9: Apply PCA on a dataset with featurenew

10: **for** *i* = 1 to 2 **do**

11:  $ND_{i}^{Train}$ ← split(*feature*_*i*_, 70%, first)

12:  $ND_{i}^{Test}$ ← split(*feature*_*i*_, 30%, last)

13: **end for**

14: Train the data with DT, RF, MLP, GNB, K-NN

15: Set the best model

16: Test the data with the best model.

17: Predict the test data

18: **if** (Class Label == *Normal*) **then**

19:  forward the packets

20: **else**

21:  Attack class classified and start to drop

22: all the subsequent requests from the source

23: **end if**

24: **End**

**Algorithm 2** Edited Nearest Neighbor-based SMOTE

1: Input: Dataset, Minority class *m*′, nearest neighbour *k*

2: Output: Balanced Dataset

3: **Begin**

4: select random data from *m*′

5: Calculate *x* = *dist*(*m*′, *k*)

6: Multiply *y* = *x* × *r* where *r* ∈ *rand* (0, 1)

7: add *y* to *m*′

8: Repeat step 5-7 until the required number of minority class is achieved.   ▹ End of SMOTE

9: set *k*, find K-nearest neighbor of the observation (*c*) and return the majority class *c*_*m*_.     ▹ Start of ENN

10: **if** *c* ≠ *c*_*m*_ **then**

11:  observation and its K nearest neighbor are removed.

12: **end if**

13: Repeat step 10 and 11 until the desired proportion of each class is fulfilled.

14: **End**

In the third phase PCA is used to address the issues related to multicollinearity, over fitting, and dimension reduction. The fourth phase uses machine learning models for training and the fifth stage is for testing and selecting the optimal model. Algorithm 1 summarizes the proposed detection model, and the SMOTE-ENN process is discussed in Algorithm 2.

## 5 Experimentation analysis

### 5.1 Simulation setup

In this paper, all experiments were performed over a machine having given configurations such as CPU Intel Core i7, 512 GHz,8 GB RAM. Python 3.9 Anaconda and Jupyter Notebook IDE are used as other additional packages. For the experiment purpose, we consider the Mininet version 2.1.0 framework and POX controller. POX can control hundreds of OpenFlow-enabled base station nodes with a flexible programming network control interface for the end-users. As opposed to NS3 and Opnet, the Mininet can easily create a virtual SDN environment with several end hosts, switches, and controllers on the Linux kernel. A tree topology has been considered which consists of 8 switches, 63 hosts, and a controller. For experimentation purposes, the hosts are treated as IoT devices that communicate with each other through edge devices through OpenFlow switches. We considered a similar test bed used in [35]. Among the IoT devices randomly one is considered as an attacker and another one is the victim.

The Table 2 presents the 15 features selected using Sequential Feature Selection (SFS) for both the KDDCup99 and CIC-IoT23 datasets. The MI score is used to evaluate the importance of each feature. Then we took 10 common features for both datasets respectively. For both the datasets, most important features were selected. These results suggest that the most important features for distinguishing between normal and anomalous traffic in both datasets are related to the network traffic itself, such as the number of bytes transferred, the number of connections, and the error rate. Additionally, the features related to the source and destination hosts are also important, such as the number of connections to the same host and the error rate for connections to the same host. These findings have implications for the development of intrusion detection systems (IDSs). By focusing on the most important features, IDSs can more effectively detect anomalous traffic and reduce false alarms.

**Table 2:** Selected features for FSMI model with both datasets.

Selected features for FSMI model with KDDCup 99 dataset				
SFS	∩	MI	⇒	Top Ten Selected Features
service		protocol_type		service
src_bytes		service		
count		flag		
rerror_rate		count		
dst_host_count		srv_count		protocol_type
dst_host_srv_count		same_srv_rate		flag
dst_host_same_src_port_rate		dst_host_serror_rate		src_bytes
dst_host_srv_diff_host_rate		diff_srv_rate		srv_count
dst_host_serror_rate		serror_rate		dst_bytes
flag		dst_host_serror_rate		dst_host_srv_count
dst_byte		dst_host_srv_count		dst_host_srv_rate
urgent		dst_host_same_src_port_rate		dst_host_serror_rate
num_failed_logins		src_bytes		dst_host_same_src_port_rate
protocol_type		dst_bytes		
srv_count		dst_host_count		
Selected features for FSMI model with CIC-IoT23 dataset				
SFS	∩	MI	⇒	Top Ten Selected Features
flow_duration		flow_duration		flow_duration
Header_Length		min		
Protocol_Type		Header_Length		
Duration		syn_count		Header_Length
Rate		Tot sum		Protocol_Type
Srate		Rate		Duration
Drate		Number		Rate
fin_flag_number		AVG	
max		Tot size		AVG
rst_flag_number		IAT		IAT
syn_count		magnitude		Number
AVG		Radius		covariance
IAT		covariance		syn_count
covariance		variance		

### 5.2 Performance measures and parameter settings

The performance of the detection model is measured using the metrics which is listed in Table 3. The confusion matrix is a function of True Positive (TP), True Negative (TN), False Positive (FP), and False Negative(FN).

**Table 3:** Performance metrics for the model.

Performance measure	Formula
Accuracy	$\frac{TP+TN}{TP+TN+FP+FN}$
Precision	$\frac{TP}{TP+FP}$
Recall	$\frac{TP}{TP+FN}$
F1-score	$2\times \frac{Precision\times Recall}{Precision+Recall}$

Table 4 provides information on the parameter settings for the used ML approaches. Each row indicates a different model, while the columns provide the parameters and values for each model. For example, the RF model employs a ‘gini’ criterion, has a maximum depth of 10, a random state of 42, and uses 10-fold cross-validation.

**Table 4:** Parameter settings of different models.

Models	Parameter Settings
RF	Criterion: gini, max_depth = 10, random_state = 42, cross validation = 10
DT	max_depth = 15, min_sample_split = 20, min sample leaf = 5, cross validation = 10
GNB	prior probability = yes, random_state = 42, verbose = 2, cross validation = 10
KNN	n_neighbour = 5, p = 5, n_sample_fit = 42, n_jobs = 1, cross validation = 10
MLP	max_iter = 42, random_state = 42, verbose = 2, cross validation = 10

### 5.3 Results and analysis

The problem of class imbalance has a significant impact on the classification algorithm’s accuracy. This massively increases the computational complexity of the DDoS solutions, making it inappropriate for usage in a real-world situation [36]. Both binary and multiclass classification can be used on the KDDCup99 and CIC-IoT23 dataset, however, we have considered multiclass classification.

#### 5.3.1 Class imbalance analysis

When ML models train over imbalanced datasets, the models can often suffer from biased learning and poor performance due to the mismatch between the class distribution and the distribution of the training data. This is because the majority class can dominate the learning process and the model can ignore the minority class, leading to poor prediction performance for minority class samples. To overcome such issues classical SMOTE technique is usually used which generates synthetic samples for the minority/majority class in order to balance the class distribution. SMOTE has the tendency to create synthetic samples that are very close to the existing minority class samples. This can lead to over-sampling bias in the resulting balanced dataset. SMOTE-ENN solves this problem by removing samples that are considered noisy or redundant after oversampling with SMOTE. In Table 5 discusses both datasets comparisons on 11 and 7 classes (out of 23 and out of 33) classes after over sampling. After applying SMOTE, we can observe that the classes are equal. However, after applying SMOTE-ENN, it removes a few synthetic samples which will help to reduce the complexity of the model. Hence, in this work, we have considered the SMOTE-ENN technique for balancing the dataset.

**Table 5:** Number of Instances (before and after balancing).

	CIC-IoT23				
No of Class	Name of All Attacks	Number of Attacks			Name of the Group in Attacks
		Before SMOTE	After SMOTE	After SMOTE-ENN	
0	ACK Fragmentation, UDP Flood, SlowLoris, ICMP Flood, RSTFIN Flood, PSHACK Flood, HTTP Flood, UDP Fragmentation, ICMP Fragmentation, TCP Flood, SYN Flood, SynonymousIP Flood	173777	173777	173777	DDoS
1	TCP Flood, HTTP Flood, SYN Flood, UDP Flood	41276	173777	173699	DoS
2	Ping Sweep, OS Scan, Host Discovery, Vulnerability Scan, Port Scan,	1860	173777	167731	Recon
3	Sql Injection, Command Injection, Backdoor Malware, Uploading Attack, XSS, Browser Hijacking	137	173777	164761	Web-Based
4	Dictionary Brute Force	63	173777	173654	Brute Force
5	Arp Spoofing, DNS Spoofing	2539	173777	172152	Spoofing
6	GREIP Flood, Greeth Flood, UDPPlain	13435	173777	171190	Mirai
**KDDCup99 Dataset**					
0	Smurf	280790	280790	280790	DoS
1	neptune	107201	280790	280710	DoS
2	normal	97277	280790	280705	Normal
3	back	2203	280790	280702	DoS
4	satan	1589	280790	280712	Probe
5	ipsweep	1247	280790	280701	Probe
6	portsweep	1040	280790	280100	Probe
7	warezclient	1020	280790	280360	R2L
8	teardrop	979	280790	280171	DoS
9	pod	264	280790	280150	DoS
10	nmap	231	280790	280090	Probe

The approximate proportions of attack classes in the KDD dataset has been changed significantly after using SMOTE and SMOTE-ENN. The proportion of different classes (DoS, Normal, Probe and R2L attacks) in the original dataset are in the ratio 79.30: 19.66: 0.83: 0.21. Similarly, the proportion of different classes in CICIoT dataset (DDoS, DoS, Recon, Web-Based, Brute Force attacks, Spoofing and Mirai) in the original are in the ratio 74.55: 17.71: 0.80: 0.06, 0.03: 1.09: 5.76. After using SMOTE and SMOTE-ENN, all classes were adjusted to have approximately equal proportions.

Further, we have shown the malicious attack detection accuracy results obtained when tested with the benchmark dataset with the proposed model. Later we show the results of various network performance evaluation metrics with SMOTE-ENN techniques. Feature selection and reduction techniques are analyzed using ML models with and without SMOTE technique. The accuracy of the models with SMOTE provides promising results but the recall of the minority class is less, i.e. the model is more dependent on the majority class. After applying SMOTE-ENN the dataset split into training and testing with the ratio of 70: 30. Different classification methods are also employed, including DT, RF, MLP, GNB, and K-NN. This methods are used to recognize and learn about DDoS attacks.

#### 5.3.2 Impact of features on model performance

**Fig 5:**
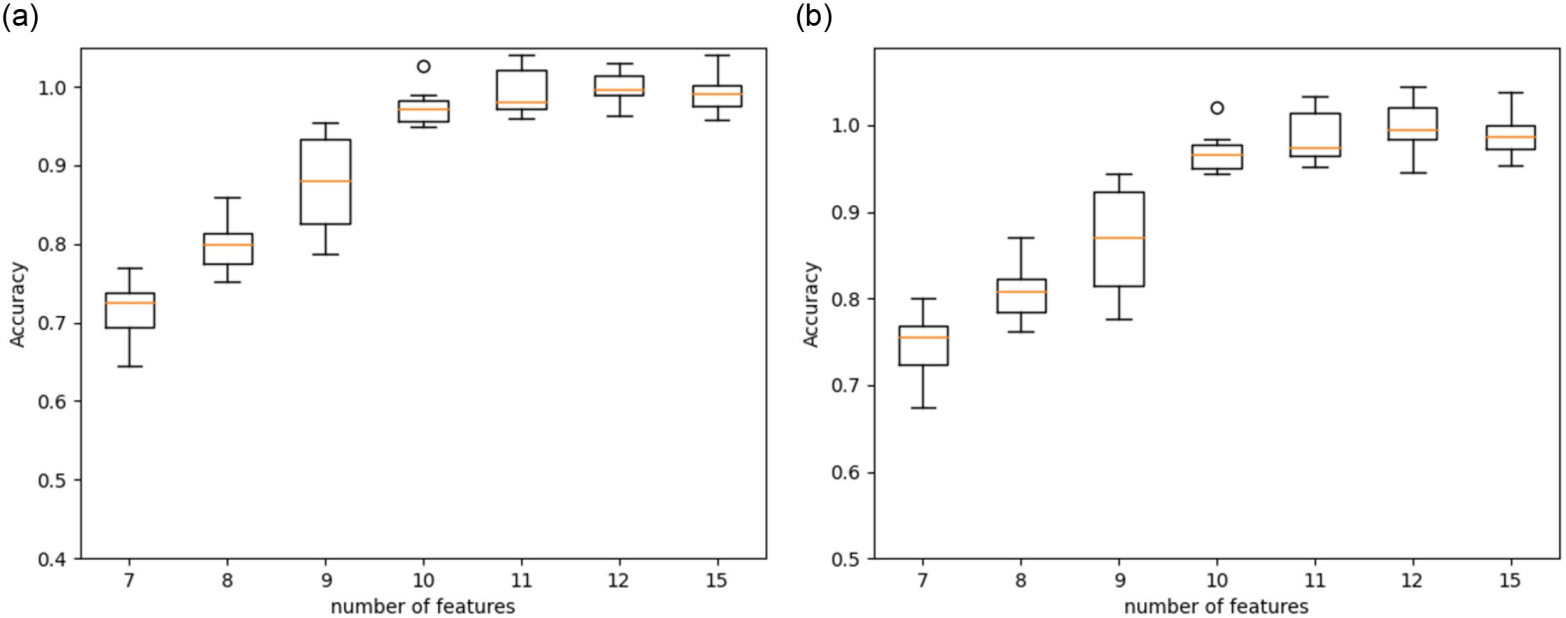
Results of accuracy with increased features. (**a**) CIC-IoT-2023 dataset, (**b**) NSL KDD99 dataset.

The balance between the simplicity of the model and accuracy was achieved in our experimental analysis by properly choosing 10 features. Choosing 15 or more features increased the accuracy, but at the same time added computing overhead and redundancy. The goal of selecting 10 features was to create a model that is accurate, comprehensible, and broadly applicable. By utilizing only 10 features, the model sacrifices only a minimal fraction of accuracy, and such marginal compromise is acceptable for resource constraint IoT enabled smart application. Fig 5a and 5b shows the Whisker plot drawn against the number of features and the accuracy using the RF classifier. It presents the minimum and maximum accuracy obtained for an increasing number of features. Features have been selected using combined SFMI and model trained with RF classifier. It can be observed that the accuracy value of the model is an acceptable range when the number of features is more than ten.

In the both datasets there are 42 and 47 features, including the target value present in the KDDCup99 and CIC-IoT datasets respectively.Therefore, we evaluate the model with top 20, 15, and 10 sets of features out of 42 and 47 features. The selected features are listed in Table 6.

**Table 6:** Selected features and their description.

**Features using SFMI (KDD-Cup)**	**Description of Features**	**Features using BFE (KDD-Cup)**	**Description of the Features**
count	number of connections to the same host as the current connection in the past two seconds	duration	length (number of seconds) of the connection
Services	network service on the destination	Services	network service on the destination
src_bytes	number of data bytes from source to destination	src_bytes	number of data bytes from source to destination
dst_bytes	number of data bytes from destination to source	dst_bytes	number of data bytes from destination to source
rerror_rate	% of connections that have ‘REJ’ errors	lnum_root	logarithm of the number of root accesses or administrative commands executed
dst_host_count	count of connections having the same destination host IP address	diff_srv_rate	% of connection to different services
dst_host_srv_count	count of connections having the same destination host IP address and service	dst_host_diff_srv_rate	rate of connection with different services than the previous connection to the same destination
dst_host_same_src_port_rate	rate of connections to the same destination host with the same source port as the current connection	dst_host_same_src_port_rate	rate of connections to the same destination host with the same source port as the current connection
dst_host_src_diff_host_rate	rate of connections to different hosts with the same source host as the current connection	dst_host_srv_diff_host_rate	rate of connections to different hosts with the same source host as the current connection
dst_host_serror_rate	percentage of connections that have an ‘SYN’ error	dst_host_serror_rate	percentage of connections that have a ‘SYN’ error
**Features using SFS (CIC-IoT23)**	**Description of Features**	**Features using BFE (CIC-IoT23)**	**Description of the Features**
flow duration	Duration of the packet’s flow	flow duration	Duration of the packet’s flow
Header_Length	Header Length	Duration	Time-to-Live(ttl)
Protocol_Type	IP, UDP, TCP, IGMP, ICMP(Integers)	Protocol_Type	IP, UDP, TCP, IGMP, ICMP(Integers)
Rate	Rate of packet transmission in a flow	ece_flag_number	Ece flag value
fin_count	Number of packets with fin flag set in the same flow	rst_count	Number of Packets with rst flag set in the same flow
urg_count	Number of packets with urg flag set in the same flow	Tot Sum	Summation of packets length in flow
HTTPs	Indicates if the application layer protocol is HTTPS	Tot size	Packet’s length
Min	Minimum packet length in the flow	Radius	Variance of the lengths of incoming packets in the flow
IAT	The time difference with the previous packet	IAT	Time difference with the previous packet
weight	Number of incoming packets * Number of outgoing packets	ack_count	Number of packets with ack flag set in the same flow

#### 5.3.3 Analysis of proposed SFMI feature selection

The common features selected by SFMI are likely to be the most informative for the model, as they have been validated by two different selection criteria. This method helps in achieving a well-performing and generalizable model.

For a comparison purpose, first we employed SFMI with and without SMOTE-ENN for both datasets in Tables 7 and 8. Accordingly, precision, recall, F1-score, and accuracy were calculated. SFMI with SMOTE-ENN has been evaluated on five different models. Among these methods, the RF obtained better accuracy i.e. 98.09% (without SMOTE-ENN) and 99.79% (with SMOTE-ENN) and GNB attained the lowest accuracy of 93.42% (without SMOTE-ENN) and 92.12% (with SMOTE-ENN) respectively with 10 features in KDD99 dataset. In the CIC-IoT 2023 dataset, the RF obtained better accuracy i.e. 99.45% (without SMOTE-ENN) and 99.95% (with SMOTE-ENN) and GNB attained the lowest accuracy with 10 features. From the tables, it can be noted that adding extra features has minimal effect on the overall accuracy. Except for GNB, all ML models perform better in the classification task. The results indicate the common features between the two methods having a higher level of consistency.

**Table 7:** Evaluation of SFMI without SMOTE-ENN (in %).

KDD-Cup99 dataset with SFMI without SMOTE-ENN (in %)						
#features	Models	DT	RF	MLP	GNB	K-NN
Feature-41	Accuracy	96.95	98.99	94.80	87.18	92.34
	Precision	96.45	97.71	94.24	91.54	90.64
	Recall	97.33	98.16	97.12	76.56	87.12
	F1-Score	96.41	97.63	92.31	89.34	91.32
Features-20	Accuracy	95.87	96.94	94.63	92.12	89.02
	Precision	97.73	97.73	88.56	84.24	90.63
	Recall	96.78	96.78	90.42	83.09	90.08
	F1-Score	96.78	97.18	88.18	85.12	90.45
Features-15	Accuracy	96.55	96.89	94.56	93.76	91.34
	Precision	98.93	98.34	89.45	86.14	89.56
	Recall	96.61	97.84	92.08	89.09	92.09
	F1-Score	96.78	97.89	89.34	89.21	91.09
Features-10	Accuracy	97.82	**98.09**	96.14	93.42	89.32
	Precision	96.17	**97.42**	92.23	91.56	92.29
	Recall	97.15	**98.53**	89.09	88.05	91.43
	F1-Score	97.33	**97.54**	90.09	90.05	91.13
Results of CIC-IoT2023 data with SFMI without SMOTE-ENN (in %)						
Features-46	Accuracy	99.37	99.34	94.57	49.208	98.79
	Precision	85.07	99.88	95.44	72.18	99.21
	Recall	99.33	82.41	95.03	49.62	99.08
	F1-score	82.17	99.64	95.23	49.08	99.21
Features-20	Accuracy	99.35	99.43	98.05	69.61	94.61
	Precision	99.35	100	99.09	86.44	95.24
	Recall	99.02	99.65	99.14	72.86	95.12
	F1-score	99.14	99.12	99.31	71.06	95.62
Features-15	Accuracy	99.32	99.41	98.56	61.07	96.51
	Precision	99.19	100	99.08	78.41	97.61
	Recall	99.39	99.29	99.21	51.26	97.05
	F1-score	99.07	99.17	99.22	52.06	97.13
Features-10	Accuracy	99.31	**99.45**	98.84	71.52	97.22
	Precision	99.13	**100**	99.93	71.47	97.51
	Recall	99.88	**99.69**	99.44	72.23	97.42
	F1-score	99.43	**99.17**	99.18	70.18	97.86

**Table 8:** Evaluation of SFMI with SMOTE-ENN (in %).

KDD-Cup99 Dataset						
Features	Models	DT	RF	MLP	GNB	K-NN
Feature-41	Accuracy	98.95	99.96	98.61	94.18	98.86
	Precision	97.95	98.08	92.03	92.25	94.93
	Recall	97.22	98.99	88.06	83.16	93.12
	F1-Score	98.05	99.58	91.05	92.64	95.76
Features-20	Accuracy	98.96	99.09	96.15	98.25	96.84
	Precision	97.48	99.37	97.06	91.03	96.12
	Recall	98.33	99.72	96.07	92.04	96.08
	F1-Score	98.03	99.39	94.07	94.04	91.08
Features-15	Accuracy	98.94	99.58	98.42	94.36	95.82
	Precision	98.09	99.17	90.45	91.14	94.34
	Recall	98.06	99.22	93.08	91.04	94.09
	F1-Score	98.08	99.05	89.08	91.09	93.09
Features-10	Accuracy	98.91	**99.79**	98.95	92.12	90.91
	Precision	98.18	**99.65**	93.23	90.08	91.89
	Recall	98.11	**98.22**	85.09	94.05	96.23
	F1-Score	98.11	**98.87**	91.07	90.05	94.23
CIC-IoT2023 Dataset						
Features-46	Accuracy	99.37	99.36	98.72	49.208	94.57
	Precision	82.01	99.73	71.46	72.49	95.85
	Recall	85.64	82.36	71.901	49.88	95.32
	F1-score	83.58	85.09	70.38	45.95	95.70
Features-20	Accuracy	99.31	99.38	98.81	50.66	95.25
	Precision	99.38	99.01	99.89	89.17	96.13
	Recall	99.08	99.05	99.08	57.08	95.61
	F1-score	99.29	99.41	99.15	55.81	95.44
Features-15	Accuracy	99.29	99.39	98.79	57.98	96.77
	Precision	99.37	99.09	99.33	66.09	97.53
	Recall	99.66	99.28	99.27	68.56	97.11
	F1-score	99.25	99.14	99.72	65.78	97.21
Features-10	Accuracy	99.27	**99.95**	98.69	51.28	97.52
	Precision	99.94	**99.32**	99.23	87.64	98.01
	Recall	99.49	**99.07**	99.57	45.06	98.52
	F1-score	99.05	**99.43**	99.65	76.43	98.51

The experimental analysis of BFE with SMOTE are detailed in Table 9. Like the previous scenario, in this experiment BFE with five different classifiers were examined. We found that SFMI performs slightly better than BFE in many cases. In few scenarios BFE outperforms the proposed method. For instance, with different feature sets, the GNB shows better performance using BFE. While with 10 features, DT and RF show comparatively better results with the proposed feature selection method.

**Table 9:** Evaluation of BFE with SMOTE-ENN (in %).

KDD-Cup99 Dataset						
#features	Models	DT	RF	MLP	GNB	K-NN
Feature-41	Accuracy	99.35	99.33	98.55	94.10	98.76
	Precision	97.88	98.71	92.53	92.58	94.81
	Recall	98.42	98.16	88.93	84.45	96.00
	F1-Score	98.59	99.00	92.25	93.04	95.01
Features-20	Accuracy	99.17	99.17	99.28	98.15	98.44
	Precision	98.33	99.35	97.13	91.11	97.10
	Recall	99.11	99.55	96.15	92.44	98.01
	F1-Score	99.00	99.33	94.05	94.13	96.88
Features-15	Accuracy	99.11	99.00	99.01	94.64	98.27
	Precision	98.11	98.45	91.66	92.41	95.33
	Recall	98.18	99.12	93.77	94.14	94.00
	F1-Score	97.08	98.11	91.08	90.08	94.99
Features-10	Accuracy	98.55	97.17	98.71	95.05	96.91
	Precision	97.18	98.65	92.77	91.51	90.27
	Recall	97.66	97.28	88.48	93.85	96.63
	F1-Score	97.11	97.91	92.09	90.15	94.07
CIC-IoT2023 Dataset						
Features-46	Accuracy	97.37	98.30	97.12	72.81	95.02
	Precision	81.22	95.14	94.55	77.11	96.15
	Recall	87.63	81.37	77.17	55.72	97.22
	F1-score	88.55	86.78	75.91	71.58	94.90
Features-20	Accuracy	98.29	98.18	98.11	68.66	96.25
	Precision	97.38	98.81	98.89	91.71	95.73
	Recall	98.51	98.65	98.48	77.28	94.61
	F1-score	98.26	99.11	98.95	75.91	94.74
Features-15	Accuracy	99.16	98.89	98.33	61.86	95.53
	Precision	98.32	98.11	98.08	77.77	96.13
	Recall	98.66	98.17	98.44	75.39	96.08
	F1-score	95.45	97.17	98.92	77.57	96.56
Features-10	Accuracy	99.01	99.19	98.22	55.20	96.62
	Precision	98.98	99.15	98.13	89.33	97.91
	Recall	99.19	98.66	98.35	62.63	96.22
	F1-score	98.65	98.44	98.59	82.33	98.81

#### 5.3.4 PCA analysis after SFMI

The dataset contains numerous features and multidimensional classes. PCA is utilized to locate the dataset’s most important attributes and makes the dataset simple. In the further experiment PCA is being utilized with SMOTE-ENN, and it is observed that it attains better results in terms of recall and F1- score. For instance, the precision and recall values of GNB were 86% and 89%, respectively without PCA with 15 features selected using SFMI (Table 10). A similar observation for MLP and K-NN. After applying PCA a better outcome was observed for GNB, KNN and MLP models. Based on the results and analysis, it can be noticed that SMOTE-ENN+SFMI with PCA performed well on all models. After comparison of all performance results, SMOTE-ENN+SFMI with PCA on the Random Forest classifier yields promising results in predicting attack traffic for all sets of features. Hence for oversampling the dataset SMOTE-ENN is being utilized, SFMI as the feature selection technique and DT is selected as the network classifier.

**Table 10:** Evaluation of SMOTE-ENN+SFMI+PCA (in %).

KDD-Cup99 Dataset using SMOTE-ENN+SFMI+PCA						
#features	Models	DT	RF	MLP	GNB	K-NN
Feature-41	Accuracy	99.99	99.93	98.51	95.18	98.91
	Precision	98.45	98.66	94.71	95.25	94.99
	Recall	98.71	99.19	85.76	88.16	96.28
	F1-Score	99.11	99.88	92.34	94.38	95.66
Features-20	Accuracy	99.01	99.11	98.11	98.15	98.01
	Precision	97.55	98.87	97.11	91.33	98.68
	Recall	98.72	96	96.18	93.76	98.11
	F1-Score	97.76	98.7	94.1	94.44	98.88
Features-15	Accuracy	99	99.09	99.06	95.65	98.62
	Precision	98.92	99.55	92.47	91.66	94.72
	Recall	97.88	98.11	95.18	93.55	97.33
	F1-Score	99	99.11	91.08	93.01	95.02
Features-10	Accuracy	98.99	**99.91**	98.95	96.44	98.9
	Precision	98.11	**99.11**	93.44	89.18	92.8
	Recall	97.11	**98.62**	89.09	95.05	96.16
	F1-Score	95	**98.99**	90.11	91.55	94.66
CIC-IoT2023 Dataset using SMOTE-ENN+SFMI+PCA						
Features-46	Accuracy	99.94	99.97	98.87	77.19	98.13
	Precision	99.56	100	96.17	96.72	98.73
	Recall	99.12	99.34	98.41	87.11	96.64
	F1-score	99.01	99.63	97.98	88.56	99.69
Features-20	Accuracy	99.83	99.96	97.15	71.15	98.82
	Precision	99.33	100	97.65	80.52	98.22
	Recall	100	99.71	98.36	79.55	99.56
	F1-score	87.91	99.53	98.13	81.43	99.16
Features-15	Accuracy	99.82	99.93	96.77	70.65	98.79
	Precision	99.27	100	97.07	78.38	99.53
	Recall	99.75	99.88	97.63	75.43	98.08
	F1-score	99.31	99.54	97.19	75.44	98.93
Features-10	Accuracy	99.83	**99.97**	97.15	70.55	97.46
	Precision	99.38	**99.39**	97.52	84.14	98.29
	Recall	100	**99.56**	97.69	67.99	97.61
	F1-score	99.01	**99.21**	98.48	80.65	98.41

The overall detection effect of each classical models on the dataset is shown in the Table 11. It is observed that the accuracy of the proposed model is roughly 1.5% superior than other models.

**Table 11:** Results comparison with classical machine learning method.

Algorithms	Precision	Recall	F1_Score	Accuracy	Features
DT (Information Gain) [10]	87.1	93.06	89.13	84.40	10
KNN [30]	77.13	99	99	98.01	10
DMLP [28]	100	100	98.91	88.19	10
RF (GINI) [47]	81.42	99.69	89.64	98.76	10
GNB [49]	92.13	91.88	91.89	80.95	10
**SMOTE-ENN+SFMI+PCA+RF(KDD-Cup)**	99.65	98.96	99.07	99.91	10
**SMOTE-ENN+SFMI+PCA+RF(CIC-IoT)**	99.97	99.39	99.56	99.21	10

The benefits of the suggested model (SFMI+PCA) are listed below.

1. The SFMI+PCA leads to a higher capability to detect DDoS attacks. For instance with 10 features GNB achieved 92.12% accuracy, whereas after PCA it achieved 96.44% in KDD-Cup dataset. In CIC-IoT dataset, DT attained 99.29% and 99.82% accuracy before and after PCA respectively.
2. By finding the best relevant features and eliminating multicollinearity concerns, the SFMI+PCA model decreases the classifier’s computational complexity.
3. Experiments reveal that the proposed FS with PCA achieves precision 99.39% with only ten features in CIC-IoT dataset whereas only SFMI achieved 99.30%.

#### 5.3.5 Multiclass classification and analysis

**Fig 6:**
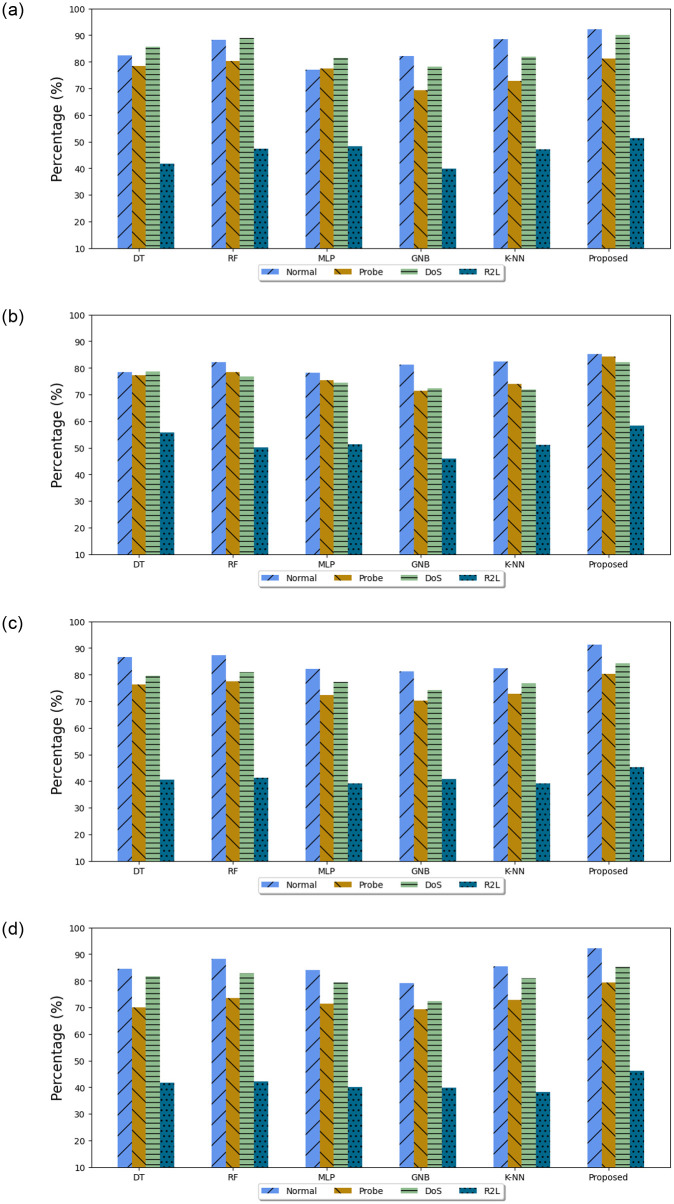
Multiclass comparison of algorithms on test data for KDDCup 99. (**a**) Accuracy, (**b**) Precision, (**c**) Recall, (**d**) F1-Score.

In Fig 6a and 6b the experimental results on the KDDCup99 test data illustrate that the proposed method has a comparatively high accuracy and precision value on all types of attack types. It has a significant advantage over other classical ML models with no feature selection mechanism. The model obtained the recall of 91.19%, 80.33%, 84.16%, and 45.37% on normal, probe, DoS, and R2L respectively. Using the proposed model, the recall value of R2L attack type was 45.56%, which was slightly higher than the classical RF model as illustrated in Fig 6c. In Fig 6d shows the statistical outcome of the F1-score metrics for the four class types in the test dataset. The model presented in this work obtained the highest F1-score of 85.16% for DoS attacks, 79.33% for Probe traffic. Compared with other classical methods, F1 results of all classes greatly improved as shown in Fig 6d.

**Fig 7:**
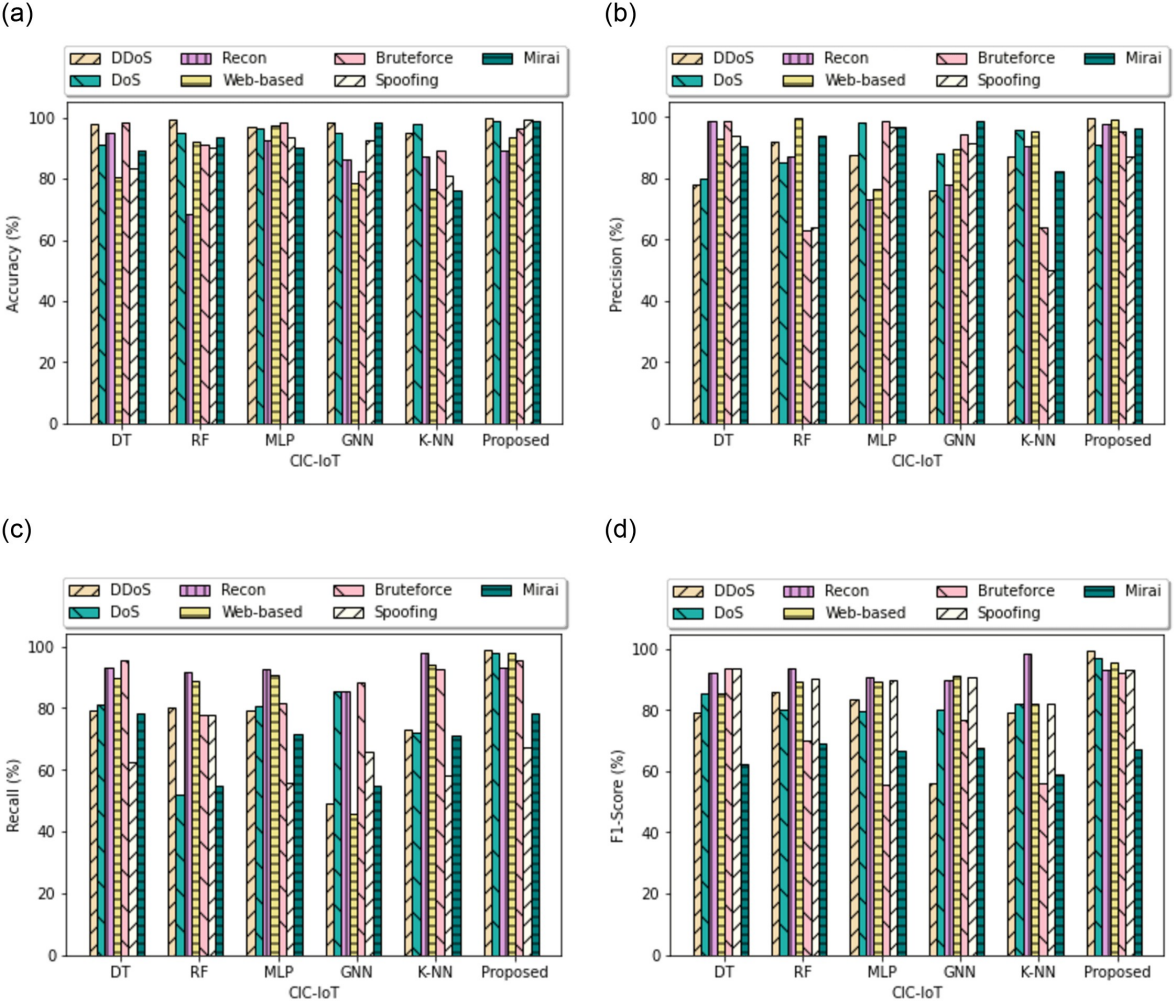
Multiclass comparison of algorithms on test data for CIC-IoT23. (**a**) Accuracy, (**b**) Precision, (**c**) Recall, (**d**) F1-Score.

In Fig 7a and 7b the experimental results on the CIC-IoT test dataset. The model obtained the recall of 99.01%, 98.13%, 93.52%, 98.23%, 65.52%, 68.00%, and 77.09% on DDoS, DoS, Recon, Brute Force, Mirai, Web-based, and Spoofing respectively. Compared with the classical RF model, there is a significant improvement in precision score in all types of attacks in both datasets. Using the proposed model, the recall value of the attack type was slightly higher than the classical RF model as illustrated in Fig 7c. In Fig 7d shows the statistical outcome of the F1-score metrics for the seven class types in the test dataset. The F1 score focuses on the recall and precision value, which exhibit the efficacy of the proposed work. So, it is obvious that there is a major impact of dataset balancing on the overall performance of the model.

#### 5.3.6 Comparative analysis with other FS techniques

**Fig 8:**
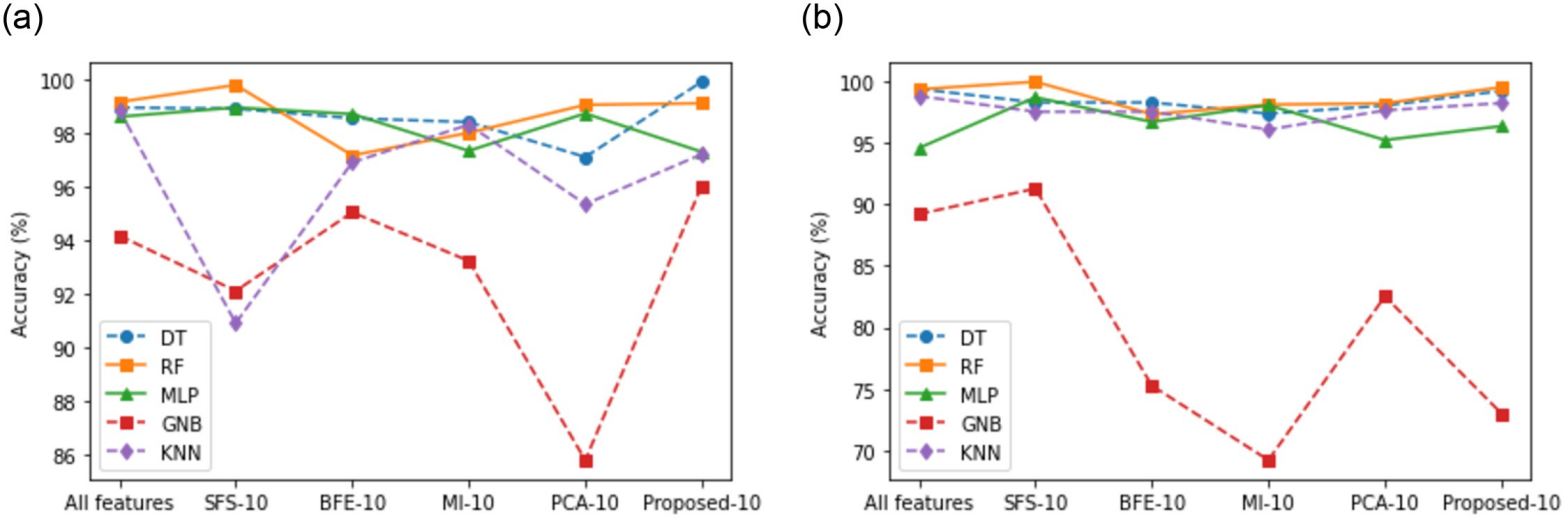
Comparison between proposed FS vs classical FS. (**a**) KDD Cup99, (**b**) CIC-IoT 2023.

It is important to observe the performance of the proposed FS approach against the standard feature selection methods. The impact of four FS methods, including our proposed approach, is visually depicted in Fig 8. To provide a comprehensive comparison, we evaluated well-established techniques such as BFE [41], SFS [15], MI [39], and PCA [40]. Fig 8a and 8b present line plots illustrating the performance of such FS techniques and their corresponding accuracies on two distinct datasets. Notably, the utilization of combined SFMI with a trained RF classifier demonstrated superior results in most of the cases. The introduced FS method significantly boosts the classifier’s performance and accuracy.

#### 5.3.7 Computational complexity

An evaluation on testing time has been made over the proposed method and compare it without FS method. Since the training phase has been made offline, however, we compare the time computation for all methods during the testing phase. The comparison of the execution time on test data has shown in Table 12. We observed that during the testing phase, the time consumption is significantly reduced with the proposed FS technique. For instance, the suggested model with feature selection achieves an average execution time of 5.34 seconds on the CIC-IoT2023 dataset, which is 35% faster than the original model (8.12 seconds) without feature selection. Comparatively, the suggested feature-selected model on the KDD-Cup99 dataset attains an average execution time of 9.21 seconds, which is 76% quicker than the initial feature-free model (38.46 seconds). These outcomes show how well the suggested feature selection technique works to increase the effectiveness of intrusion detection systems.

**Table 12:** Testing time (Sec) of different models with feature selection.

Models	Without FS (CIC-IoT)	With Proposed Model (CIC-IoT)	Without FS (KDD-Cup)	With Proposed Model (KDD-Cup)
DT	8.124	5.34	12.57	1.45
RF	224.29	40.496	38.46	9.21
MLP	690.54	87.632	58.56	10.23
GNB	3.858	4.732	19.67	1.57
KNN	416.53	4.012	1002.71	792.45

**Fig 9:**
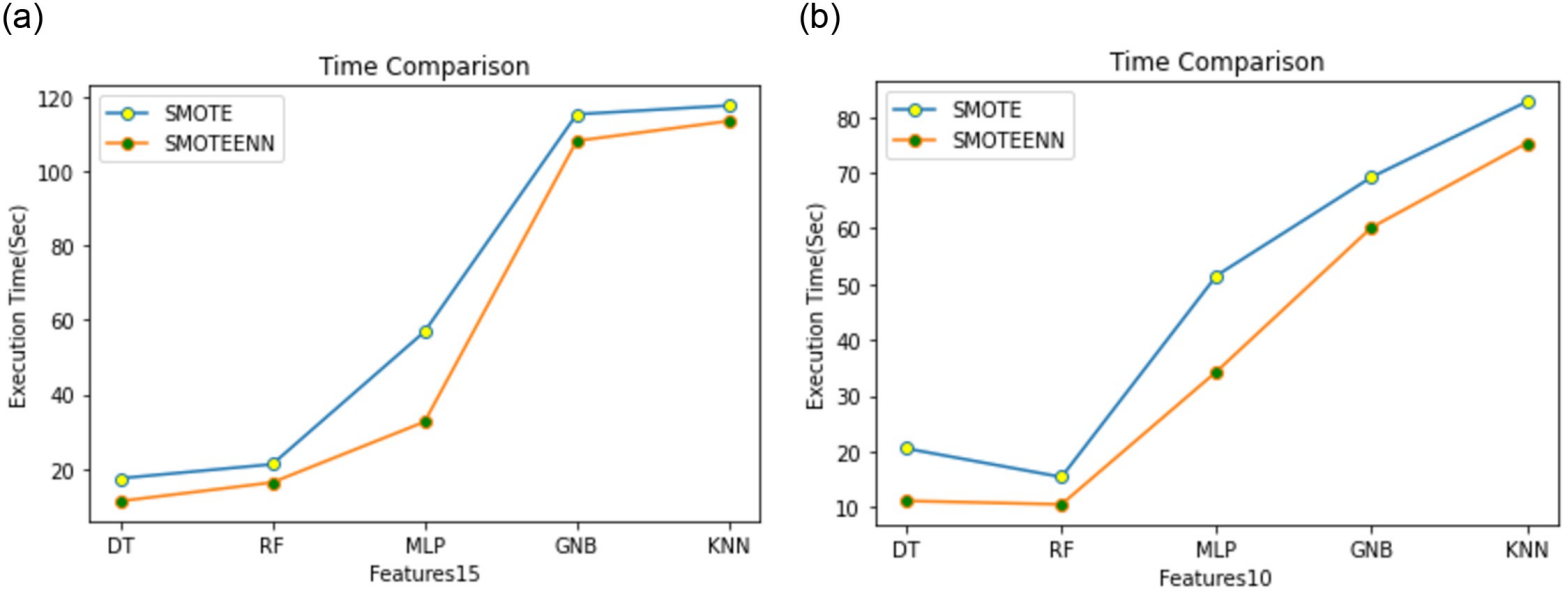
Comparison between SMOTE and SMOTE-ENN in execution time with F-15 and F-10.

In another experiment, we tested the computational complexity of the SMOTE and SMOTE-ENN techniques. From Fig 9 it is observed that SMOTE-ENN takes less execution time than the simple SMOTE technique with 10 and 15 features. SMOTE-ENN not only generates samples but also removes noisy samples and borderline examples using ENN. This cleaning process might result in a more manageable dataset for subsequent processing steps, which could help to reduce overall computation time. The usage of the SMOTE-ENN technique has been proven that it can improve the detection rate in imbalanced training data. Moreover, important features of the KDD dataset have been effectively selected by using the SFS+PCA method. Hence the SMOTE-ENN with proposed feature selection technique and RF model can be useful in the context of SDIoT.

**Fig 10:**
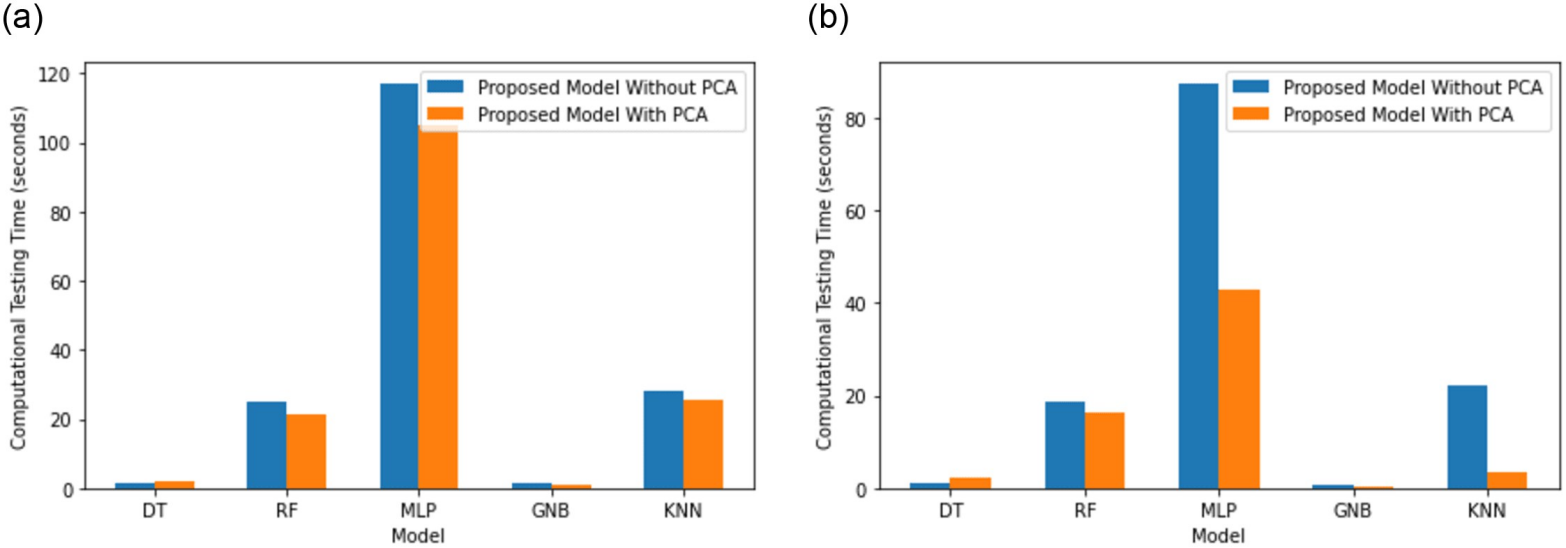
Testing time (sec) of proposed model with PCA vs without PCA. (**a**) KDD Cup99, (**b**) CIC-IoT 2023.

Fig 10 shows the testing time of PCA and without PCA for the CIC-IoT and KDD-Cup99 data sets. The SFMI+PCA model typically exhibits faster testing times than the SFMI model. The SFMI+PCA with DT model takes an average of 2.2 seconds to test on the CIC-IoT23 dataset, whereas the SFS+MI model takes an average of 0.9619 seconds. The SFMI+PCA model takes an average of 2.137 seconds to test on the KDD-Cup99 dataset, whereas the SFMI model takes an average of 1.242 seconds. This indicates a 72% increase in the SFMI model’s testing duration. The SFMI+PCA improves the model in terms of efficiency as well as accuracy. This is apparently due to PCA reducing the dimensionality of the data, which may reduce the computing time of the classification operation.

**Fig 11:**
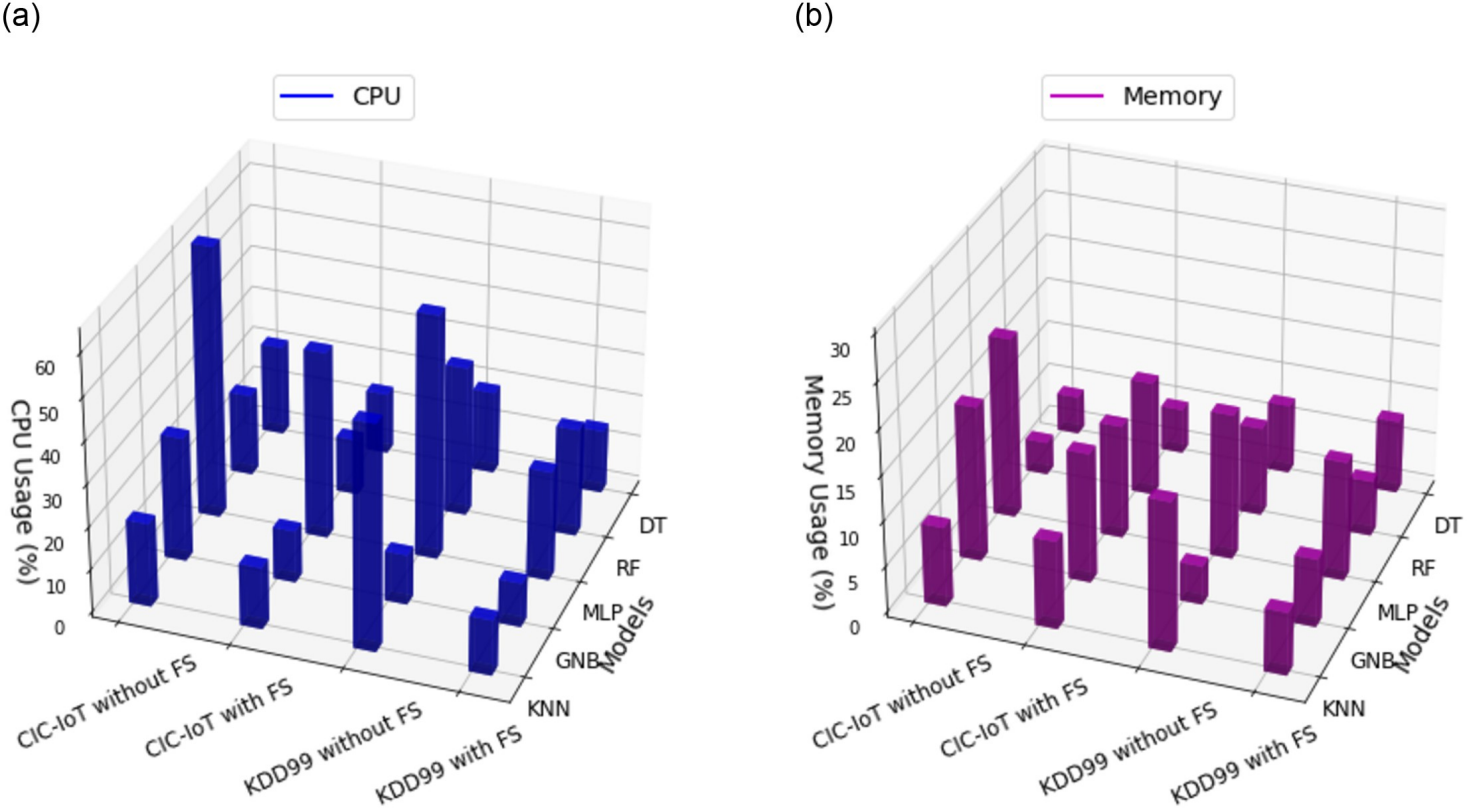
CPU and Memory usage of both the datasets. (**a**) CPU Usage, (**b**) Memory Usage.

A comparative analysis of CPU and memory usage has been made in Fig 11 Judging from graph, it can be noticed that the models’ CPU and memory usage has been reduced marginally after FS. For instance, in CIC-IoT dataset, the RF model without FS has the memory usage at 9.66%, whereas after FS it was reduced to 7.01%. The GNB model with FS has the smallest CPU utilization of 11.31%, which was 12.11% before FS. These findings highlight the importance of feature selection on CPU and memory usage across models and datasets.

## 6 Conclusion and future work

The framework is divided into five primary phases, the first of which deals with the mean value replacement technique for addressing missing values standardization and normalization. In the second phase, the author proposed the Synthetic Minority Over-sampling Technique is used to correct the data imbalance problem. Then it combines two phases from SMOTE to SMOTE-ENN. In order to solve the issue of dataset noise, we employed the ENN approach to eliminate instances of either class. Feature selection is done in the third phase utilizing the feature significance technique to reduce the computational complexity of the model. We proposed SFMI that combines the advantages of both SFE and Mutual Information techniques. Top common features were extracted from the nominated features based on SFE and MI. Then, an improved prediction framework is modeled using a combination of Decision Tree, Random Forest, Multi-layer Perceptron, Gaussian Naive Bayes, and KNN. This framework is validated using the KDDCup99 and CIC-IoT23 dataset that shows better accuracy in RF model. Thus, the proposed framework combines pre-processing using SMOTE-ENN, feature selection using SFMI and PCA techniques for the recognition of malicious attacks. The proposed model can provide various benefits to IoT applications, such as effective resource utilization, reduced downtime, reduced economic loss, and resilience against evolving threats. In future, the adaptability of the proposed model can be improved on evolving attacks using ensemble models. Moreover, the model will be tested on different benchmark datasets for its validation and performance measurement. The relevant features from multiple datasets will be studied with impactful FS techniques.
